# Occupational Contact Allergy to Components of Epoxy Resin Systems: Descriptive and Comparative Epidemiological Analyses Based on IVDK Data 2008–2022

**DOI:** 10.1111/cod.70222

**Published:** 2026-07-29

**Authors:** David Reissig, Katharina Heymann, Steffen Schubert, Richard Brans, Birger Kränke, Eva Oppel, Rüdiger Panzer, Dagmar Simon, Andrea Bauer, for the IVDK

**Affiliations:** ^1^ Department of Dermatology University Allergy Center, University Hospital Carl Gustav Carus, Technical University Dresden Dresden Germany; ^2^ Information Network of Departments of Dermatology (IVDK) Institute at the Georg‐August University Göttingen, University Medical Centre Göttingen Göttingen Germany; ^3^ Institute for Interdisciplinary Dermatologic Prevention and Rehabilitation (iDerm), Osnabrück University Osnabrück Germany; ^4^ Department of Dermatology, Environmental Medicine, and Health Theory Osnabrück University Osnabrück Germany; ^5^ Department of Dermatology and Venereology Medical University of Graz Graz Austria; ^6^ Department of Dermatology and Allergology Ludwig‐Maximilians‐University Munich Germany; ^7^ Clinic and Policlinic for Dermatology, Venereology, and Allergology University Medical Center Rostock Rostock Germany; ^8^ Department of Dermatology Bern University Hospital Bern Switzerland

## Abstract

**Background:**

Epoxy resin systems (ERS) frequently cause occupational allergic contact dermatitis.

**Objectives:**

To analyze and update sensitization frequencies to epoxy resin, reactive diluents and hardeners in patients with occupational dermatitis (OD).

**Methods:**

Retrospective descriptive and comparative analysis of data collected by the Information Network of Departments of Dermatology (IVDK) from OD patients patch tested with bisphenol A diglycidyl ether (DGEBA) epoxy resin, 2008–2022.

**Results:**

DGEBA sensitization declined overall, with fluctuations, from 4.2% in 2008–2010 to 2.9% in 2020–2022 (*p* = 0.0014). Adjusted odds ratios (ORs) were highest for plastics processors (11.7; 95% CI, 8.1–16.6), bricklayers, cement and concrete workers (6.4; 5.1–8.0), and painters/varnishers (5.3; 4.0–6.9). DGEBA‐positive patients were most frequently co‐sensitised to the reactive diluents 1,6‐hexanediol diglycidyl ether (43.6%), 1,4‐butanediol diglycidyl ether (35.7%), and phenyl glycidyl ether (30.8%), and to the hardener m‐xylylenediamine (22.7%). DGEBA testing alone would not have identified 446 patients (1.9% of all DGEBA‐tested OD patients) with sensitizations to reactive diluents or amine hardeners.

**Conclusions:**

If contact allergy to ERS components is suspected, not only DGEBA, but also reactive diluents and hardeners should be patch tested. Prevention should prioritise exposure control, occupational hygiene, ongoing surveillance, and periodic test series updates.

## Graphical abstract

This study analyzes patch test data from 23 969 patients with occupational dermatitis to characterise sensitization to bisphenol A diglycidyl ether (DGEBA), reactive diluents, and amine hardeners. The findings show an overall decline in DGEBA sensitization with fluctuations, substantial co‐sensitization, and markedly increased risks in construction‐related, plastics processing, and painting/varnishing occupations, underscoring the need for extended patch testing and targeted preventive measures.

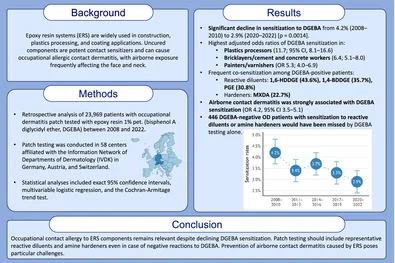

## 1 Introduction

Epoxy resin systems (ERS) comprise resins, reactive diluents, and hardeners that cure to high‐performance polymers. All of them are known to cause contact sensitization which usually arises during handling of uncured components leading to allergic contact dermatitis (ACD), mainly affecting the hands. Face and neck involvement due to airborne exposure and secondary contamination is common [1, 2, 3, 4]. ERS are widely used in construction, automotive manufacturing, aerospace, and the metal and electronics industries. They are employed as strong adhesives, durable surface coatings, for electrical insulation, and in the production of composite materials [5, 6, 7, 8, 9]. Accordingly, a high share of sensitizations to components of ERS is well documented among construction workers, painters and varnishers, plastics processors, and metalworkers, although exposure conditions and sensitization rates vary by trade [9, 10, 11, 12, 13].

Low‐molecular‐weight monomers and oligomers of epoxy resins, such as the diglycidyl ether of bisphenol A (DGEBA; CAS 1675‐54‐3) and the diglycidyl ether of bisphenol F (DGEBF; CAS 28064‐14‐4), are among the most frequent occupational contact sensitizers [8, 9, 14]. In contemporary multicenter patch test studies, the prevalence of sensitization to DGEBA is about 1%–2% in consecutive patients and 3%–6% among patients with occupational dermatitis (OD) [8, 15]. While the sensitising potential of DGEBA is well documented [5, 6, 8, 16], reactive diluents and hardeners are less comprehensively characterised [9, 10, 11, 17]. Hardeners in cold‐curing ERS are typically amines, while reactive diluents are commonly aromatic or aliphatic glycidyl ethers. Prior analyses of patch test data collected by the Information Network of Departments of Dermatology (IVDK) between 2002 and 2011 showed that nearly half of DGEBA‐sensitised patients also reacted to at least one diluent or hardener, with *m*‐xylylenediamine (MXDA; CAS 1477‐55‐0), isophorone diamine (IPDA; CAS 2855‐13‐2), 1,6‐hexanediol diglycidyl ether (1,6‐HDDGE; CAS 16096‐31‐4), and 1,4‐butanediol diglycidyl ether (1,4‐BDDGE; CAS 2425‐79‐8) featuring prominently [10, 11]. Subsequent studies have confirmed these patterns [9, 17, 18].

This study aims at updating previous IVDK data by assessing time trends in sensitization to DGEBA among OD patients and by comparing and quantifying sensitization to reactive diluents and hardeners among OD patients with or without sensitization to DGEBA as well as identifying the most affected occupational groups and suspected allergen sources, describing clinical features, including face involvement and airborne contact dermatitis.

## 2 Patients and Methods

The present analysis is based on data from 2008 to 2022 collected by the Information Network of Departments of Dermatology (IVDK), a collaboration of 58 dermatology departments in Germany, Austria, and Switzerland. The structure and routine procedures of the IVDK have been described in detail elsewhere [19]. In brief, patient histories, clinical information, and patch test results are documented locally in a standardised format and, following pseudonymization, transferred twice annually to the IVDK central office. After a standardised quality control process, data are integrated into the central IVDK database and analyzed according to international standards [20, 21].

All departments participating in the IVDK are also members of the German Contact Dermatitis Research Group (Deutsche Kontaktallergie‐Gruppe; DKG). Patch testing was performed and evaluated in accordance with the DKG guidelines [22, 23]. Patch test preparations were predominantly obtained from Almirall Hermal (Reinbek, Germany) until 2013 and from SmartPractice Europe (Greven, Germany) thereafter; in some cases, test substances were also purchased from Chemotechnique (Vellinge, Sweden). The patch test exposure time was 48 h in most patients (84.2%), while 24‐h exposure was applied in 15.8% of cases. In nearly all patients, Finn Chambers (8 mm inner diameter) on Scanpor tape were used as test chambers. For the current analysis, readings on day 3 (D3) were used. In rare cases where a reading was conducted on day 4 (D4) instead, this result was included. Only positive reactions scored as +, ++ or +++ [defined by erythema, infiltration, papules and/or (coalescing) vesicles] were classified as positive for the purpose of dichotomized analyses. In group comparisons, positive and negative reactions were used, whereas doubtful, follicular, and irritant reactions were excluded. Patch testing included the DKG baseline series and the additional DKG series for resins/adhesives. Bisphenol F epoxy resin (DGEBF, 0.25% pet.), which was never part of any DKG test series, was additionally tested in a small number of patients (*n* = 34). Patients were compared according to their patch test reaction to DGEBA (positive [sensitised] vs. negative). The present analyses focused on amine hardeners and aromatic/aliphatic glycidyl ether reactive diluents, whereas anhydride hardeners were not part of this series and were therefore not analysed. A complete list of substances, abbreviations, and CAS numbers is provided in Table S1. Concomitant positive patch test reactions between selected substance pairs were evaluated in a separate analysis of IVDK data from 2008 to 2022. This analysis included all patients with valid patch test results for both substances of the respective pair and was not restricted to the OD cohort. For 1,6‐HDDGE and 1,4‐BDDGE, the denominator was defined as all patients with a positive reaction to either one or both reactive diluents. For phenyl glycidyl ether (PGE) and DGEBA, the denominator was defined as all PGE‐positive patients who had also been tested with DGEBA.

Between 2008 and 2022, a total of 163 984 patients (170 127 consultations) were patch tested in the departments of dermatology participating in the IVDK. OD was assessed by the treating dermatologists at the participating IVDK centers and indicated within the standardised IVDK documentation. In addition, up to three suspected allergen sources and the main indication for patch testing were documented prior to patch testing. With regard to patients' medical and occupational history and the alignment of patch test results with occupational and non‐occupational exposures, patients were classified as OD cases if their dermatitis was documented as conceivably caused by their occupation. Based on this assessment, OD was documented in 27 655 patients (29 037 consultations). Patients with missing or unknown occupational relatedness were not included in the OD cohort. Analyses were conducted at the patient level; in patients with multiple consultations, the consultation with the strongest reaction to DGEBA was retained. This retrospective analysis focuses on 23 969 OD patients who were patch tested with DGEBA 1% pet. between 2008 and 2022. The MOAHLFA index items were used to describe patient characteristics. The item O referred to OD, the item A to atopic dermatitis (past or present), and the items H, L and F denoted the primarily affected anatomical sites (hand, leg and face). In line with recent IVDK reporting and the updated MOAHLFA(P) classification, the age component was defined as age ≥ 40 years [24]. Final main diagnoses, including allergic contact dermatitis, were taken from the final clinical diagnosis documented in the IVDK after completion of the diagnostic work‐up and patch testing. Allergic contact dermatitis was not inferred automatically from patch test positivity, but was based on thorough individual assessment of clinical relevance.

The statistical analyses included comparisons of reaction frequencies with surrogate exposure data, logistic regression analyses, and an exact Cochran–Armitage trend test. A multivariable logistic regression analysis was performed with a positive patch test reaction to DGEBA as the dependent variable. Explanatory variables included occupational group (indicator variables, reference ‘all other occupations’), a combined indicator for airborne contact dermatitis and/or face dermatitis, and past or current atopic dermatitis. In this context, face dermatitis refers to the MOAHLFA item F and denotes the primarily affected body site, not a specific exposure route. Airborne contact dermatitis refers to a clinically documented diagnosis (secondary diagnosis respected) in the IVDK case record in which an airborne exposure route was considered involved. The combined subgroup ‘airborne contact dermatitis and/or face dermatitis’ included patients fulfilling either criterion, including those fulfilling both.

The model was adjusted for age ≥ 40 years and for male sex. Results are presented as odds ratios (ORs) with 95% confidence intervals (95% CIs) based on profile‐likelihood estimates. Model performance was assessed using the Hosmer‐Lemeshow goodness‐of‐fit test (*p* > 0.05). There was no indication of relevant multicollinearity (all VIF < 5) and no evidence of separation. Proportions of anamnestic items and reaction frequencies in distinct patient groups are presented with 95% CIs, calculated using the Wilson score method. Group differences were considered significant when the 95% CIs did not overlap. For the Cochran–Armitage trend test, a *p*‐value < 0.05 was considered significant. Data management and statistical analyses were performed using SAS, version 9.4 (SAS Institute Inc., Cary, NC, USA) [25] and R version 4.5.1 (R Foundation for Statistical Computing, Vienna, Austria) [26] via RStudio version 2025.05.1 Build 513 [27].

## 3 Results

### 3.1 Prevalence of Sensitization to Diglycidyl Ether of Bisphenol A (DGEBA)

Overall, 23 969 patients with OD were patch tested with DGEBA during the study period (2008–2022), of whom 842 had a positive reaction to DGEBA (3.5%, 95% CI 3.3–3.8). Of the remaining patients, 22 925 had a negative reaction and 202 had doubtful, follicular, or irritant reactions, which were excluded from dichotomized group comparisons. Among the 842 positive DGEBA reactions, 392 (46.6%) were +, 332 (39.4%) were ++, and 118 (14.0%) were +++. A longitudinal analysis of DGEBA sensitization frequencies in patients with OD revealed a statistically significant downward trend with fluctuations. Sensitization declined overall from 4.2% (95% CI 3.7–4.8) in 2008–2010 to 2.9% (95% CI 2.4–3.4) in 2020–2022 (Figure 1 and Table S2). This downward trend was confirmed by the exact Cochran–Armitage test (*p* = 0.0014).

**FIGURE 1:**
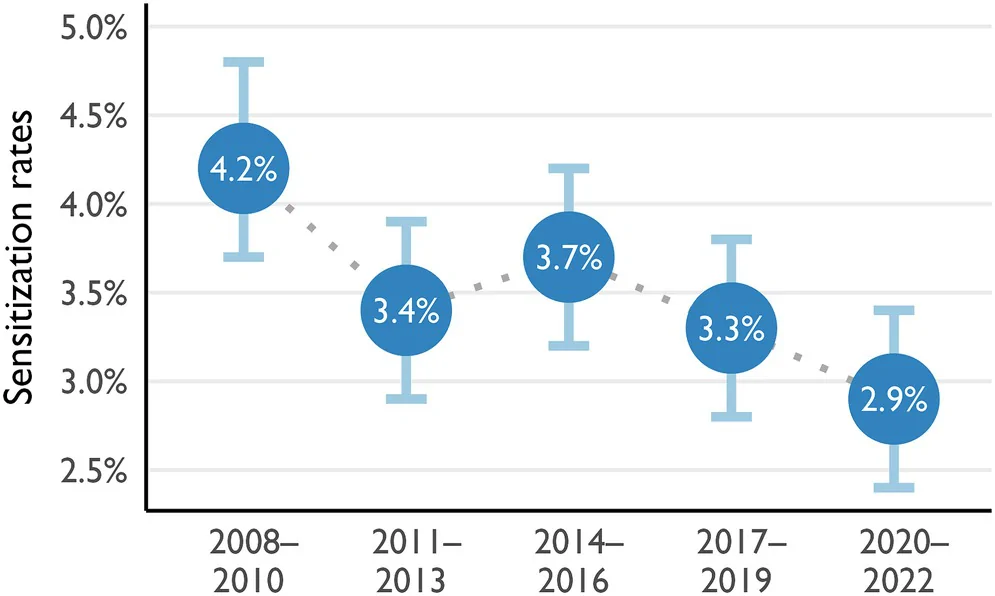
Time trends in sensitization to diglycidyl ether of bisphenol A (DGEBA) resin in patients with occupational dermatitis from 2008 to 2022. Data points represent 3‐year intervals.

### 3.2 MOAHLFA Index

Table 1 summarises demographic and clinical characteristics of the study population according to the MOAHLFA index. All items except leg dermatitis and OD showed significant differences in subgroup comparisons. Compared to DGEBA‐negative OD patients (*n* = 22 925), those with sensitization to DGEBA (*n* = 842) were significantly more often male (74.6%, 95% CI 71.5–77.4 vs. 48.8%, 95% CI 48.2–49.4), 40 years or older (63.5%, 95% CI 60.2–66.7 vs. 55.5%, 95% CI 54.8–56.1) and had face dermatitis (18.8%, 95% CI 16.3–21.5 vs. 5.0%, 95% CI 4.8–5.3). In contrast, past or current atopic dermatitis (20.3%, 95% CI 17.7–23.2 vs. 33.9%, 95% CI 33.3–34.5) and hand dermatitis (60.3%, 95% CI 57.0–63.6 vs. 82.1%, 95% CI 81.6–82.6) were significantly less frequent in OD patients with sensitization to DGEBA compared to those without.

**TABLE 1:** Demographic and clinical characteristics (MOAHLFA index) of patients with occupational dermatitis (OD) with and without sensitization to diglycidyl ether of bisphenol A (DGEBA).

	DGEBA‐positive OD patients (*N* = 842)		DGEBA‐negative OD patients (*N* = 22 925)	
	*n*	% [95% CI]	*n*	% [95% CI]
Male (M)	**628**	**74.6 [71.5–77.4]**	**11 188**	**48.8 [48.2–49.4]**
Occupational dermatitis (O)^a^	842^a^	100^a^	22 925^a^	100^a^
(past or present) Atopic dermatitis (A)	**171**	**20.3 [17.7–23.2]**	**7763**	**33.9 [33.3–34.5]**
Hand dermatitis (H)	**508**	**60.3 [57.0–63.6]**	**18 819**	**82.1 [81.6–82.6]**
Leg dermatitis (L)	9	1.1 [0.6–2.0]	156	0.7 [0.6–0.8]
Face dermatitis (F)	**158**	**18.8 [16.3–21.5]**	**1156**	**5.0 [4.8–5.3]**
Age ≥ 40 years (A)	**535**	**63.5 [60.2–66.7]**	**12 715**	**55.5 [54.8–56.1]**

Abbreviations: 95% CI, 95% confidence interval; DGEBA, diglycidyl ether of bisphenol A; OD, occupational dermatitis.

^a^ Owing to group definition. Significant differences are marked in bold.

Among the 842 OD patients with sensitization to DGEBA, 171 (20.3%) had airborne contact dermatitis and/or face dermatitis. Table S3 presents their MOAHLFA characteristics compared to those of DGEBA‐positive OD patients without airborne contact dermatitis or face dermatitis. Patients in the combined subgroup were less likely to have past or present atopic dermatitis (12.9%, 95% CI 8.7–18.7 vs. 22.2%, 95% CI 19.2–25.5). According to group definitions, marked differences were observed with regard to hand dermatitis (4.1%, 95% CI 2.0–8.2 vs. 74.7%, 95% CI 71.2–77.8) and face dermatitis (91.8%, 95% CI 86.7–95.1 vs. 0.1%, 95% CI 0.0–0.8). All other items showed non‐significant differences.

### 3.3 Final Main Diagnosis

The final main diagnoses differed markedly between OD patients with and without sensitization to DGEBA. ACD was much more frequent among DGEBA‐positive OD patients (66.0%, 95% CI 62.8–69.2 vs. 29.5%, 95% CI 28.9–30.1), as was airborne contact dermatitis (6.7%, 95% CI 5.2–8.5 vs. 1.0%, 95% CI 0.8–1.1). In contrast, irritant contact dermatitis (7.7%, 95% CI 6.1–9.7 vs. 25.1%, 95% CI 24.5–25.6), atopic dermatitis (4.5%, 95% CI 3.3–6.1 vs. 12.1%, 95% CI 11.7–12.6), acute recurrent vesicular dermatitis (3.1%, 95% CI 2.1–4.5 vs. 6.4%, 95% CI 6.1–6.7), and hyperkeratotic eczema (1.2%, 95% CI 0.6–2.2 vs. 7.0%, 95% CI 6.6–7.3) were all more common in DGEBA‐negative OD patients.

### 3.4 Occupations

Table 2 displays the most frequent occupational groups among OD patients with and without sensitization to DGEBA. Bricklayers, cement and concrete workers were markedly overrepresented among DGEBA‐positive patients (15.6%, 95% CI 13.3–18.2 vs. 2.5%, 95% CI 2.3–2.7), followed by painters and varnishers (9.9%, 95% CI 8.0–12.1 vs. 1.8%, 95% CI 1.6–2.0), plastics processors (5.7%, 95% CI 4.3–7.5 vs. 0.5%, 95% CI 0.5–0.6), engineers, technicians, architects, and mathematicians (4.0%, 95% CI 2.9–5.6 vs. 2.4%, 95% CI 2.2–2.6), and road and civil engineering workers (2.4%, 95% CI 1.5–3.6 vs. 0.4%, 95% CI 0.3–0.5). By contrast, health care professionals (e.g., nurses, midwives, physiotherapists) were substantially less common among DGEBA‐sensitised patients (2.9%, 95% CI 1.9–4.2 vs. 16.5%, 95% CI 16.0–17.0). A listing of all recorded occupational groups is provided in Table S4.

**TABLE 2:** Occupational groups with significant differences between patients with occupational dermatitis (OD) with and without sensitization to diglycidyl ether of bisphenol A (DGEBA).

	DGEBA‐positive OD patients (*N* = 842)		DGEBA‐negative OD patients (*N* = 22 925)	
	*n*	% [95% CI]	*n*	% [95% CI]
Bricklayers, cement and concrete workers	**131**	**15.6 [13.3–18.2]**	**562**	**2.5 [2.3–2.7]**
Painters, varnishers	**83**	**9.9 [8.0–12.1]**	**409**	**1.8 [1.6–2.0]**
Plastics processors	**48**	**5.7 [4.3–7.5]**	**124**	**0.5 [0.5–0.6]**
Engineers, technicians, architects, mathematicians	**34**	**4.0 [2.9–5.6]**	**539**	**2.4 [2.2–2.6]**
Health care professionals (e.g., nurses, midwives, physiotherapists)	24	2.9 [1.9–4.2]	3781	16.5 [16.0–17.0]
Road and civil engineering workers	**20**	**2.4 [1.5–3.6]**	**91**	**0.4 [0.3–0.5]**

*Note:* Only items with significant differences between both groups are displayed. Significantly higher frequencies among DGEBA‐positive OD patients are marked in bold.

Abbreviations: 95% CI, 95% confidence interval; DGEBA, diglycidyl ether of bisphenol A; OD, occupational dermatitis.

To explore occupational patterns in OD patients with airborne contact dermatitis and/or face dermatitis, a subgroup analysis was conducted among DGEBA‐positive patients. Painters and varnishers were significantly more frequent in the combined subgroup (18.1%, 95% CI 13.1–24.6 vs. 7.7%, 95% CI 6.0–10.0). All other occupational categories showed comparable distributions between the two subgroups.

To further evaluate independent risk factors of sensitization to DGEBA, a multivariable logistic regression analysis was performed, adjusted for age ≥ 40 years and male sex. Compared to all other occupations (reference), significantly increased odds ratios (ORs) of DGEBA sensitization were observed in plastics processors (OR 11.7, 95% CI 8.1–16.6), bricklayers, cement and concrete workers (OR 6.4, 95% CI 5.1–8.0), road and civil engineering workers (OR 6.3, 95% CI 3.7–10.3), and painters and varnishers (OR 5.3, 95% CI 4.0–6.9). Carpenters and woodworkers also showed an elevated odds ratio (OR 1.7, 95% CI 1.0–2.8). In addition, airborne contact dermatitis and/or face dermatitis was strongly associated with DGEBA sensitization (OR 4.2, 95% CI 3.5–5.1), whereas past or current atopic dermatitis was inversely associated (OR 0.7, 95% CI 0.5–0.8) (Table 3).

**TABLE 3:** Results of multivariable logistic regression^a^ for sensitization to diglycidyl ether of bisphenol A (DGEBA).

Predictor	OR	95% CI
Occupational group (current job)		
Bricklayers, cement and concrete workers	6.4	5.1–8.0
Painters, varnishers	5.3	4.0–6.9
Plastics processors	11.7	8.1–16.6
Road and civil engineering workers	6.3	3.7–10.3
Carpenters, woodworkers	1.7	1.0–2.8
All other occupations (reference)	1.0	
Other factors		
Airborne contact dermatitis and/or face dermatitis	4.2	3.5–5.1
Past or current atopic dermatitis	0.7	0.5–0.8

Abbreviations: 95% CI, 95% confidence interval; DGEBA, diglycidyl ether of bisphenol A; OR, odds ratio.

^a^ Adjusted for age ≥ 40 years and sex (male).

### 3.5 Main Suspected Allergen Sources

The distribution of main suspected allergen sources prior to patch testing among OD patients with and without sensitization to DGEBA is provided in Table S5. These entries were used as exposure proxies; their clinical relevance was not systematically confirmed in the present analysis, and categories were not mutually exclusive. Compared with DGEBA‐negative patients, adhesives (30.0%, 95% CI 27.0–33.2 vs. 4.9%, 95% CI 4.7–5.2), building materials (21.5%, 95% CI 18.9–24.4 vs. 5.4%, 95% CI 5.1–5.7), plastics (17.6%, 95% CI 15.2–20.3 vs. 3.4%, 95% CI 3.2–3.6), and paints/varnishes (13.7%, 95% CI 11.5–16.1 vs. 3.7%, 95% CI 3.5–3.9) were documented more often in DGEBA‐sensitised individuals. In contrast, gloves (16.9%, 95% CI 14.5–19.5 vs. 32.3%, 95% CI 31.7–32.9) and rubber (11.3%, 95% CI 9.3–13.6 vs. 14.9%, 95% CI 14.5–15.4) were documented less frequently in DGEBA‐positive patients.

To investigate whether certain exposure sources were more often associated with airborne contact dermatitis and/or face dermatitis, a subgroup analysis was conducted among DGEBA‐positive OD patients. In this subgroup, adhesives (39.2%, 95% CI 32.2–46.7 vs. 27.7%, 95% CI 24.5–31.2) and paints/varnishes (20.5%, 95% CI 15.1–27.1 vs. 11.9%, 95% CI 9.7–14.6) were documented more frequently, whereas gloves (4.7%, 95% CI 2.4–9.0 vs. 20.0%, 95% CI 17.1–23.2) and disinfectants (1.2%, 95% CI 0.3–4.2 vs. 7.5%, 95% CI 5.7–9.7) were documented less often as main suspected allergen sources. All other exposure categories showed non‐significant differences between the two subgroups.

### 3.6 Sensitization to Components of ERS

Patch test results for selected components of ERS are summarised in Table 4. Among reactive diluents, the highest frequencies of sensitizations in DGEBA‐positive OD patients were observed for 1,6‐hexanediol diglycidyl ether (1,6‐HDDGE, 43.6%, 95% CI 39.6–47.6), 1,4‐butanediol diglycidyl ether (1,4‐BDDGE, 35.7%, 95% CI 31.9–39.6), PGE (30.8%, 95% CI 27.2–34.5), and *p*‐tert‐butylphenyl glycidyl ether (PTBPGE, 28.6%, 95% CI 24.7–32.8). In a separate analysis of IVDK data from 2008 to 2022, frequent concomitant positive reactions were observed among patients tested with both substances of the respective pair. Of 580 patients with a positive reaction to either 1,6‐HDDGE or 1,4‐BDDGE, 374 reacted to both substances (64.5%, 95% CI 60.5–68.3). Among 359 PGE‐positive patients who had also been tested with DGEBA, 274 reacted to both substances (76.3%, 95% CI 71.7–80.4). In the DGEBA‐negative group, reaction rates for these diluents were uniformly low and the differences were significant: 1,4‐BDDGE 1.2% (95% CI 0.9–1.5), 1,6‐HDDGE 1.1% (95% CI 0.8–1.4), PGE 0.5% (95% CI 0.4–0.8), PTBPGE 0.2% (95% CI 0.1–0.4).

**TABLE 4:** Frequencies of patch testing and positive reactions in DGEBA‐positive and DGEBA‐negative patients with occupational dermatitis (OD), Information Network of Departments of Dermatology (IVDK), 2008–2022.

Substance	Conc. (vehicle)	DGEBA‐positive OD patients (*n* = 842)				DGEBA‐negative OD patients (*n* = 22 925)				
		*n* tested	*n* positive	% positive	95% CI	*n* tested	*n* positive	% positive	95% CI	
Reactive diluents										
1,6‐Hexanediol diglycidyl ether (1,6‐HDDGE)	0.25% pet.	**617**	**269**	**43.6**	**39.6–47.6**	**5193**	**57**	**1.1**	**0.8–1.4**	
1,4‐Butanediol diglycidyl ether (1,4‐BDDGE)	0.25% pet.	**614**	**219**	**35.7**	**31.9–39.6**	**5127**	**59**	**1.2**	**0.9–1.5**	
Phenyl glycidyl ether (PGE)	0.25% pet.	**637**	**196**	**30.8**	**27.2–34.5**	**6480**	**35**	**0.5**	**0.4–0.8**	
*p*‐*tert*‐Butylphenyl glycidyl ether (PTBPGE)	0.25% pet.	**496**	**142**	**28.6**	**24.7–32.8**	**4250**	**8**	**0.2**	**0.1–0.4**	
Cresyl glycidyl ether (CGE)	0.25% pet.	**616**	**94**	**15.3**	**12.5–18.3**	**5066**	**20**	**0.4**	**0.2–0.6**	
Butyl glycidyl ether (BGE)	0.25% pet.	**618**	**73**	**11.8**	**9.4–14.6**	**5197**	**25**	**0.5**	**0.3–0.7**	
Trimethylolpropane triglycidyl ether (TMPTGE)	0.25% pet.	**575**	**46**	**8.0**	**5.9–10.5**	**4921**	**6**	**0.1**	**0.0–0.3**	
Hardeners										
*m*‐Xylylene diamine (MXDA)	0.10% pet.	**576**	**131**	**22.7**	**19.4–26.4**	**4938**	**38**	**0.8**	**0.5–1.1**	
4,4′‐Diaminodiphenylmethane (MDA)	0.50% pet.	**638**	**90**	**14.1**	**11.5–17.1**	**6826**	**189**	**2.8**	**2.4–3.2**	
Isophorone diamine (IPDA)	0.50% pet.	**623**	**56**	**9.0**	**6.9–11.5**	**5241**	**24**	**0.5**	**0.3–0.7**	
2,4,6‐Tris(dimethylaminomethyl)phenol (tris‐DMP)	0.50% pet.	**178**	**14**	**7.9**	**4.4–12.8**	**1657**	**2**	**0.1**	**0.0–0.4**	
Trimethylhexane‐1,6‐diamine (TMHDA)	0.50% pet.	**577**	**37**	**6.4**	**4.6–8.7**	**4907**	**29**	**0.6**	**0.4–0.8**	
Diethylenetriamine (DETA)	1% pet.	**611**	**26**	**4.3**	**2.8–6.2**	**5155**	**56**	**1.1**	**0.8–1.4**	
Triethylenetetramine (TETA)	0.50% pet.	134	4	3.0	0.8–7.5	961	4	0.4	0.1–1.1	
Ethylenediamine dihydrochloride (EDHCl)	1% pet.	525	4	0.8	0.2–1.9	15 806	73	0.5	0.4–0.6	
Other epoxy‐related substances										
Bisphenol F epoxy resin (DGEBF)	0.25% pet.	**34**	**21**	**61.8**	**43.6–77.8**		**163**	**1**	**0.6**	**0.0–3.4**
Bisphenol A‐glycidyl methacrylate (BIS‐GMA)	2% pet.	**568**	**110**	**19.4**	**16.2–22.9**		**4532**	**19**	**0.4**	**0.3–0.7**

*Note:* For CAS numbers, see Table S1. Significant differences are marked in bold.

Abbreviations: 95% CI, 95% confidence interval; OD, occupational dermatitis; pet., petrolatum.

Among hardeners, the most frequent positive reactions in DGEBA‐positive patients were observed for m‐xylylenediamine (MXDA, 22.7%, 95% CI 19.4–26.4) and 4,4′‐diaminodiphenylmethane (MDA, 14.1%, 95% CI 11.5–17.1). In the DGEBA‐negative group, reaction frequencies were markedly lower (MXDA 0.8%, 95% CI 0.5–1.1; MDA 2.8%, 95% CI 2.4–3.2). Differences between groups were significant for all hardeners except TETA and EDHCl.

Among DGEBA‐positive patients, co‐reactivity with bisphenol A‐glycidyl methacrylate (BIS‐GMA) was observed in 19.4% (95% CI 16.2–22.9), and with bisphenol F diglycidyl ether epoxy resin (DGEBF) in 61.8% (95% CI 43.6–77.8) of those tested (*n* = 34). In the DGEBA‐negative group, reaction frequencies were 0.4% (95% CI 0.3–0.7) for BIS‐GMA and 0.6% (95% CI 0.0–3.4) for DGEBF. A subgroup analysis of DGEBA‐positive patients stratified by airborne contact dermatitis and/or face dermatitis showed largely similar sensitization frequencies across all tested patch test preparations (Table S6).

## 4 Discussion

This large multicenter study characterised OD patients with sensitization to DGEBA regarding population characteristics and patterns of concomitant patch test reactivity to other components of ERS. Over the study period, DGEBA sensitization was frequent, but showed a downward trend with fluctuations in OD patients. DGEBA sensitization was associated with specific exposures such as adhesives and building materials which are predominantly used by various occupational groups such as construction workers or painters and varnishers. Concomitant sensitization to reactive diluents and hardeners was common and not only found in DGEBA‐positive OD patients, necessitating additional patch testing beyond DGEBA in the baseline series. The frequent occurrence of face dermatitis and airborne contact dermatitis has implications for preventive measures.

### 4.1 Time Trends in DGEBA Sensitization

DGEBA sensitization declined overall from 4.2% in 2008–2010 to 2.9% in 2020–2022, with a significant downward trend and fluctuations between periods. This pattern is in accordance with findings from a North American hospital revealing a decrease of positivity to DGEBA when tested in the baseline series in the period from 2007 to 2016 [28]. Earlier IVDK reports placed overall DGEBA positivity at 1.1%–1.7% before 2008 and at 1.3%–1.9% in 2012–2015, while within the OD subset rates declined to 2.7% in 2015–2017 and rose to 3.9% in 2018, indicating temporal variability within Germany, Austria and Switzerland [5, 18]. External European reports are heterogeneous, as they show increasing epoxy resin sensitization in Italian construction workers [29], but stable sensitization rates in Greek construction workers [30]. European registry data on patients with OD indicate that epoxy resin sensitization remains relevant as an occupational allergen [8].

From a surveillance perspective, the observed decline in DGEBA sensitization should be interpreted cautiously. The registry‐based design does not allow causal attribution of this trend. Although improved exposure control in certain sectors, for example by automation with closed systems, standardised mixing procedures, more appropriate glove use, formulation changes, and component substitution, have been reported and may have contributed to reduced exposure and subsequently lower DGEBA sensitization frequencies [4, 18, 31, 32], our data cannot distinguish these explanations from other influences, including temporal changes in the tested OD population, changes in the relative contribution of participating centers, possible shifts in diagnostic or documentation practices, and variation inherent to patch test surveillance data. Thus, the decreasing DGEBA sensitization frequency should be considered a descriptive finding. These considerations argue for continuous surveillance, including periodic re‐evaluation of the test series, e.g., whether DGEBA should be patch tested together with hardeners and reactive diluents to avoid underestimation of sensitizations and to guide targeted prevention [18, 28, 31, 32].

### 4.2 Sociodemographic Characteristics and Clinical Presentation

OD patients with sensitization to DGEBA exhibited a characteristic clinical profile. Compared with DGEBA‐negative OD patients, they were more often male and more frequently presented with airborne contact dermatitis and/or face dermatitis, whereas hand dermatitis and past or current atopic dermatitis were less common. This distribution may partly reflect the male predominance in epoxy‐exposed trades and selection mechanisms, including both avoidance and early discontinuation of occupations with a high skin strain by atopic workers, consistent with a healthy worker effect. The frequent occurrence of face dermatitis and airborne contact dermatitis suggests that relevant exposure may extend beyond direct hand contact, including airborne exposure and indirect transfer from contaminated hands during epoxy work. In line with previous reports, DGEBA sensitization is overrepresented among airborne contact dermatitis cases, and face dermatitis is associated with DGEBA positivity [3, 12]. Prevention of airborne contact dermatitis caused by ERS poses particular challenges and may adversely affect prospects of continued employment in exposed trades, which underlines the need for early recognition and consistent exposure control.

### 4.3 Occupational Groups at Risk of DGEBA Sensitization

Marked differences in prevalence of DGEBA sensitization among the various occupations were evident after adjustment for age and sex, with the highest odds ratios in plastics processors and elevated odds in construction‐related occupations, painters and varnishers. These patterns are consistent with the main suspected allergen sources, which cluster in adhesives, building chemicals, plastics, and paints/varnishes, and are in accordance with earlier IVDK analyses and reports from Italy, Greece, and North America [5, 12, 13, 29, 30].

A limitation of the risk assessments is that the job title ‘plastics processors’ used in the registry is not well‐defined and may also capture workers who actually perform epoxy‐related tasks in the construction industry (building trade). This may lead to overestimation of the associated risk for this occupational group [1]. Conversely, classic chemical industry settings tend to report few epoxy allergies, likely reflecting better hygiene standards and greater use of automation and closed systems, resulting in minimal direct skin contact and airborne exposure [33]. These considerations do not affect the main message that construction and plastics‐related activities remain priority targets for prevention, but they underline the need to interpret occupation fields together with concrete exposure scenarios and task descriptions [32, 34].

### 4.4 Sensitization to Reactive Diluents, Hardeners, and Other Epoxy‐Related Substances

In our study population of patients with OD and sensitization to DGEBA, concomitant sensitizations to reactive diluents, including 1,6‐HDDGE, 1,4‐BDDGE, PGE, and PTBPGE, as well as to hardeners, including MXDA, MDA, and IPDA, were frequent, whereas rates among DGEBA‐negative patients were consistently low. These findings align with earlier IVDK data and data of the Finnish Institute of Occupational Health (FIOH) showing that concomitant sensitizations to aliphatic diglycidyl ethers, such as 1,6‐HDDGE and 1,4‐BDDGE, as well as MXDA are frequent among OD patients sensitised to DGEBA [1, 10, 11].

Frequent co‐sensitization to both 1,6‐HDDGE and 1,4‐BDDGE was observed in our study, which may be explained by their close structural similarity, although immunological cross‐reactivity has not been formally demonstrated. In contrast, both our data and experimental studies confirm the strong link between DGEBA and PGE. Cross‐reactivity between them has been observed in animal models [35]. This may also explain the frequent positive reactions to 1,4‐BDDGE, which is only rarely used in Germany, and to PGE, which is no longer in use in ERS. Cresyl glycidyl ether (CGE) and PTBPGE are still used in current applications [9, 10, 11, 16]. Cross‐reactivity has also been described between PGE and CGE, whereas PTBPGE appears to act as an independent allergen [1, 10].

With regard to hardeners, positive reactions to MDA must be interpreted with caution. This compound has not been employed as a hardener in ERS for many years, and positive tests most plausibly indicate cross‐reactivity to other para‐compounds or primary exposure to 4,4′‐diphenylmethane diisocyanate (MDI) with subsequent cutaneous conversion to MDA rather than current occupational exposure to MDA itself [36].

Beyond diluents and hardeners, concomitant reactions were also observed to other epoxy‐related substances, notably DGEBF and BIS‐GMA, with markedly higher reaction rates among DGEBA‐positive patients compared to DGEBA‐negative patients. For DGEBF, concomitant reactions with DGEBA are frequent and may result from immunological cross‐reactivity or co‐exposure [37]. With regard to BIS‐GMA, epidemiological data suggest possible immunological cross‐reactivity with DGEBA [38].

In suspected epoxy resin allergy or in the presence of relevant occupational exposure, patch testing should not be limited to DGEBA, which is often the only ERS marker included in the baseline series. Among DGEBA‐negative patients, isolated sensitizations were observed to 1,6‐HDDGE (1.1%, 95% CI 0.8–1.4), 1,4‐BDDGE (1.2%, 95% CI 0.9–1.5), CGE (0.4%, 95% CI 0.2–0.6), MXDA (0.8%, 95% CI 0.5–1.1), and IPDA (0.5%, 95% CI 0.3–0.7). These observations indicate that a certain subset of patients reacts only to these components. Overall, approximately 31% of all positive patch test reactions to reactive diluents and amine hardeners occurred in DGEBA‐negative patients. At least one positive reaction to reactive diluents or amine hardeners was noted in 446 DGEBA‐negative patients (1.9% of all DGEBA‐tested OD patients). This number represents 115 patients with at least one positive reaction to a reactive diluent and 360 patients with at least one positive reaction to an amine hardener, with an overlap of 29 patients who reacted to both substance groups. Positive reactions only to DGEBF or BIS‐GMA were not included in this number. This underscores that surveillance relying exclusively on DGEBA can underestimate sensitization to ERS components. Representative reactive diluents such as 1,6‐HDDGE, 1,4‐BDDGE, or CGE, and amine hardeners such as MXDA and IPDA should routinely be included. Where feasible, workplace materials should be added, in particular when screening with the commercial test preparations is negative but the clinical suspicion of ACD persists [1].

From a prevention perspective, workplace measurements document relevant DGEBA residues on hands, face, tools, and work surfaces during mixing and application of ERS and during subsequent cleaning procedures [4, 13, 29]. Evidence from a cohort of workers in the wind‐turbine industry and a German case–control study in construction workers indicates that current epoxy exposure, work practices, and insufficient protective equipment are major determinants of the risk of occupational ACD [31, 32]. Prevention should therefore prioritise exposure control at these critical steps and ensure selection and correct use of suitable chemical‐resistant gloves together with effective hygiene measures [31, 32].

### 4.5 Limitations

Test panels and availability of commercial test preparations varied across centers and years. In addition, temporal trends derived from registry data may be influenced by changes in the tested population, the relative contribution of participating centers, diagnostic and documentation practices, and the limitations of patch test surveillance data. Therefore, the observed decline in DGEBA sensitization should not be interpreted causally. Patch‐test readings were predominantly performed until day 3 and therefore later reactions have possibly been missed [39]. Not all currently used constituents of ERS were included, and patients' own workplace materials were not systematically tested, so under‐ascertainment of sensitizations to certain allergens is possible. Anhydride hardeners were not part of the routinely used test series and were therefore not assessed; results cannot be generalised to anhydrides.

## 5 Conclusions

Sensitization to components of ERS in patients with OD is driven mainly by DGEBA, glycidyl ether reactive diluents, and amine hardeners. The highest odds for DGEBA sensitization were observed among plastics processors, bricklayers, cement and concrete workers, and painters/varnishers. Although DGEBA sensitization declined over time, sensitization to components of ERS remains an important occupational problem, and this descriptive trend should continue to be monitored. Baseline screening with DGEBA alone may fail to detect all patients sensitised to ERS because a subset reacts only to hardeners or reactive diluents. Routine patch testing in suspected cases should therefore include representative hardeners and reactive diluents and, where available, patients' own workplace materials. Prevention should focus on minimising skin and airborne exposure during mixing, application, and cleanup of ERS and on the correct selection and use of chemical‐resistant gloves, with targeted measures in the identified high‐risk occupations. Continued surveillance with periodic updates to the test series is needed to ensure accurate monitoring and to guide prevention.

## Author Contributions

**David Reissig:** conceptualization, methodology, software, investigation, validation, formal analysis, visualization, writing – original draft. **Katharina Heymann:** conceptualization, investigation, formal analysis, visualization. **Steffen Schubert:** conceptualization, methodology, software, data curation, investigation, validation, formal analysis, resources. **Richard Brans:** writing – review and editing. **Birger Kränke:** writing – review and editing. **Eva Oppel:** writing – review and editing. **Rüdiger Panzer:** writing – review and editing. **Dagmar Simon:** writing – review and editing. **Andrea Bauer:** conceptualization, methodology, supervision, writing – review and editing.

## Consent

The authors have nothing to report.

## Conflicts of Interest

The IVDK, maintained by the IVDK e.V., of which S. Schubert is an employee, is sponsored by the chemical, cosmetic and fragrance industry (associations) as well as by public funds. For details, see http://ivdk.org/en/sponsors/. All other authors declare no conflicts of interest.

## Supporting information


**Table S1:** Test substances used for patch testing, abbreviations, CAS numbers, substance class, and test concentrations.
**Table S2:** Time trend of sensitization to diglycidyl ether of bisphenol A (DGEBA) in patients with occupational dermatitis.
**Table S3:** Demographic and clinical characteristics (MOAHLFA index) of patients with occupational dermatitis (OD) and sensitization to diglycidyl ether of bisphenol A (DGEBA), stratified by airborne contact dermatitis and/or face dermatitis.
**Table S4:** Occupational groups of patients with occupational dermatitis (OD) with and without sensitization to diglycidyl ether of bisphenol A (DGEBA).
**Table S5:** Main suspected allergen sources in patients with occupational dermatitis (OD) with and without sensitization to diglycidyl ether of bisphenol A (DGEBA).
**Table S6:** Frequencies of patch testing and positive reactions in DGEBA‐positive patients with occupational dermatitis (OD), stratified by airborne contact dermatitis and/or face dermatitis.

## Data Availability

Data sharing is not possible due to legal restrictions under applicable European data protection law.
