# Supplementary material for: Occupational Contact Allergy to Components of Epoxy Resin Systems: Descriptive and Comparative Epidemiological Analyses Based on IVDK Data 2008–2022
Source: Contact Dermatitis. 2026 Jul 29;95(5):482–91. doi: 10.1111/cod.70222 (PMC13648310; doi:10.1111/cod.70222)
Supplement: Supplementary file 1 [file COD-95-482-s001.docx]

# Supplementary Tables and Figures

Table S1. Test substances used for patch testing, abbreviations, CAS numbers, substance class, and test concentrations.

| Substance | Abbreviation | CAS No. | Substance class | Conc. (vehicle) |
| --- | --- | --- | --- | --- |
| 1,6-Hexanediol diglycidyl ether | 1,6-HDDGE | 16096-31-4 | reactive diluent | 0.25% pet. |
| 1,4-Butanediol diglycidyl ether | 1,4-BDDGE | 2425-79-8 | reactive diluent | 0.25% pet. |
| Phenyl glycidyl ether | PGE | 122-60-1 | reactive diluent | 0.25% pet. |
| p-tert-Butylphenyl glycidyl ether | PTBPGE | 3101-60-8 | reactive diluent | 0.25% pet. |
| Cresyl glycidyl ether | CGE | 2210-79-9 | reactive diluent | 0.25% pet. |
| Butyl glycidyl ether | BGE | 2426-08-6 | reactive diluent | 0.25% pet. |
| Trimethylolpropane triglycidyl ether | TMPTGE | 30499-70-8 | reactive diluent | 0.25% pet. |
| m-Xylylene diamine | MXDA | 1477-55-0 | hardener | 0.10% pet. |
| 4,4′-Diaminodiphenylmethane | MDA | 101-77-9 | hardener | 0.50% pet. |
| Isophorone diamine | IPDA | 2855-13-2 | hardener | 0.50% pet. |
| 2,4,6-Tris(dimethylaminomethyl)phenol | tris-DMP | 90-72-2 | hardener | 0.50% pet. |
| Trimethylhexane-1,6-diamine | TMHDA | 25620-58-0 | hardener | 0.50% pet. |
| Diethylenetriamine | DETA | 111-40-0 | hardener | 1% pet. |
| Triethylenetetramine | TETA | 112-24-3 | hardener | 0.50% pet. |
| Ethylenediamine dihydrochloride | EDHCl | 333-18-6 | hardener | 1% pet. |
| Bisphenol F epoxy resin | DGEBF | 9003-36-5; 28064-14-4 | epoxy resin | 0.25% pet. |
| Bisphenol A-glycidyl methacrylate | BIS-GMA | 1565-94-2 | methacrylate | 2% pet. |

Abbreviations: pet., petrolatum.

Table S2. Time trend of sensitization to diglycidyl ether of bisphenol A (DGEBA) in patients with occupational dermatitis.

| Period | n tested | n positive | % positive | 95% CI |
| --- | --- | --- | --- | --- |
| 2008 – 2022 (Total) | 23969 | 842 | 3.5 | 3.3 – 3.8 |
| 2008 – 2010 | 4923 | 208 | 4.2 | 3.7 – 4.8 |
| 2011 – 2013 | 5429 | 183 | 3.4 | 2.9 – 3.9 |
| 2014 – 2016 | 5124 | 189 | 3.7 | 3.2 – 4.2 |
| 2017 – 2019 | 4504 | 148 | 3.3 | 2.8 – 3.8 |
| 2020 – 2022 | 3989 | 114 | 2.9 | 2.4 – 3.4 |

Abbreviation: 95% CI, 95% confidence interval.

Table S3. Demographic and clinical characteristics (MOAHLFA index) of patients with occupational dermatitis (OD) and sensitization to diglycidyl ether of bisphenol A (DGEBA), stratified by airborne contact dermatitis and/or face dermatitis.

|  | DGEBA-positive OD patients with airborne contact dermatitis and/or face dermatitis  (N = 171) | | DGEBA-positive OD patients without airborne contact dermatitis or face dermatitis  (N = 671) | |
| --- | --- | --- | --- | --- |
|  | n | % [95% CI] | n | % [95% CI] |
| Male (M) | 132 | 77.2 [70.3 – 82.8] | 496 | 73.9 [70.5 – 77.1] |
| Occupational dermatitis (O)* | 171* | 100* | 671* | 100* |
| Atopic dermatitis (A) | **22** | **12.9 [8.7 – 18.7]** | **149** | **22.2 [19.2 – 25.5]** |
| Hand dermatitis (H) | **7** | **4.1 [2.0 – 8.2]** | **501** | **74.7 [71.2 – 77.8]** |
| Leg dermatitis (L) | 0 | 0.0 [0.0 – 2.2] | 9 | 1.3 [0.7 – 2.5] |
| Face dermatitis (F) | **157** | **91.8 [86.7 – 95.1]** | **1** | **0.1 [0.0 – 0.8]** |
| Age ≥ 40 years (A) | 102 | 59.6 [52.2 – 66.7] | 433 | 64.5 [60.8 – 68.1] |

*Owing to group definition. Significant differences are marked in bold. Abbreviations: DGEBA, diglycidyl ether of bisphenol A; OD, occupational dermatitis; 95% CI, 95% confidence interval.

Table S4. Occupational groups of patients with occupational dermatitis (OD) with and without sensitization to diglycidyl ether of bisphenol A (DGEBA).

|  | DGEBA-positive OD patients  (N = 842) | | DGEBA-negative OD patients  (N = 22,925) | |
| --- | --- | --- | --- | --- |
|  | n | % [95% CI] | n | % [95% CI] |
| Bricklayers, cement and concrete workers | **131** | **15.6 [13.3 – 18.2]** | **562** | **2.5 [2.3 – 2.7]** |
| Occupations with unspecified exposure | **124** | **14.7 [12.5 – 17.3]** | **1,870** | **8.2 [7.8 – 8.5]** |
| Mechanics, machine operators, toolmakers | 93 | 11.0 [9.1 – 13.3] | 2,672 | 11.7 [11.2 – 12.1] |
| Painters, varnishers | **83** | **9.9 [8.0 – 12.1]** | **409** | **1.8 [1.6 – 2.0]** |
| Plastics processors | **48** | **5.7 [4.3 – 7.5]** | **124** | **0.5 [0.5 – 0.6]** |
| Engineers, technicians, architects, mathematicians | **34** | **4.0 [2.9 – 5.6]** | **539** | **2.4 [2.2 – 2.6]** |
| Health care professionals (e.g., nurses, midwives, physiotherapists) | **24** | **2.9 [1.9 – 4.2]** | **3,781** | **16.5 [16.0 – 17.0]** |
| Sheet metal workers, fitters, metalworkers | 23 | 2.7 [1.8 – 4.1] | 504 | 2.2 [2.0 – 2.4] |
| Missing or incorrect entry | **22** | **2.6 [1.7 – 3.9]** | **324** | **1.4 [1.3 – 1.6]** |
| Road and civil engineering workers | **20** | **2.4 [1.5 – 3.6]** | **91** | **0.4 [0.3 – 0.5]** |
| Carpenters, woodworkers | 18 | 2.1 [1.4 – 3.4] | 279 | 1.2 [1.1 – 1.4] |
| Cleaners (buildings, vehicles, streets, etc.) | **17** | **2.0 [1.3 – 3.2]** | **1,047** | **4.6 [4.3 – 4.8]** |
| Electricians | 14 | 1.7 [1.0 – 2.8] | 418 | 1.8 [1.7 – 2.0] |
| Chemists, chemical lab technicians, chemical workers | 13 | 1.5 [0.9 – 2.6] | 353 | 1.5 [1.4 – 1.7] |
| Metal shapers (cutting) | **13** | **1.5 [0.9 – 2.6]** | **964** | **4.2 [4.0 – 4.5]** |
| Office workers | 12 | 1.4 [0.8 – 2.5] | 548 | 2.4 [2.2 – 2.6] |
| Housewives, domestic caregivers | 9 | 1.1 [0.6 – 2.0] | 310 | 1.4 [1.2 – 1.5] |
| Warehouse, transport, shipping workers, goods inspectors | 8 | 1.0 [0.5 – 1.9] | 336 | 1.5 [1.3 – 1.6] |
| Dentists | 8 | 1.0 [0.5 – 1.9] | 379 | 1.7 [1.5 – 1.8] |
| Gardeners | **6** | **0.7 [0.3 – 1.5]** | **404** | **1.8 [1.6 – 1.9]** |

Significant differences are marked in bold. Abbreviations: DGEBA, diglycidyl ether of bisphenol A; OD, occupational dermatitis; 95% CI, 95% confidence interval.

Table S5. Main suspected allergen sources in patients with occupational dermatitis (OD) with and without sensitization to diglycidyl ether of bisphenol A (DGEBA).

|  | DGEBA-positive OD patients  (N = 842) | | DGEBA-negative OD patients  (N = 22,925) | |
| --- | --- | --- | --- | --- |
|  | n | % [95% CI] | n | % [95% CI] |
| Adhesives | **253** | **30.0 [27.0 – 33.2]** | **1,129** | **4.9 [4.7 – 5.2]** |
| Building materials | **181** | **21.5 [18.9 – 24.4]** | **1,236** | **5.4 [5.1 – 5.7]** |
| Plastics | **148** | **17.6 [15.2 – 20.3]** | **781** | **3.4 [3.2 – 3.6]** |
| Gloves | **142** | **16.9 [14.5 – 19.5]** | **7,411** | **32.3 [31.7 – 32.9]** |
| Paints, varnishes | **115** | **13.7 [11.5 – 16.1]** | **847** | **3.7 [3.5 – 3.9]** |
| Rubber (other) | **95** | **11.3 [9.3 – 13.6]** | **3,426** | **14.9 [14.5 – 15.4]** |
| Cosmetics, creams, sunscreens | **83** | **9.9 [8.0 – 12.1]** | **4,827** | **21.1 [20.5 – 21.6]** |
| Metalworking fluids | **67** | **8.0 [6.3 – 10.0]** | **3,239** | **14.1 [13.7 – 14.6]** |
| Chemicals (other) | **52** | **6.2 [4.7 – 8.0]** | **898** | **3.9 [3.7 – 4.2]** |
| Disinfectants | **52** | **6.2 [4.7 – 8.0]** | **5,815** | **25.4 [24.8 – 25.9]** |
| Metals (processing) | **40** | **4.8 [3.5 – 6.4]** | **1,724** | **7.5 [7.2 – 7.9]** |
| Cleaning agents | **30** | **3.6 [2.5 – 5.0]** | **1,557** | **6.8 [6.5 – 7.1]** |
| Topical medications | **25** | **3.0 [2.0 – 4.3]** | **1,075** | **4.7 [4.4 – 5.0]** |
| Paint fumes / fresh paint | **25** | **3.0 [2.0 – 4.3]** | **164** | **0.7 [0.6 – 0.8]** |
| Fats, oils (non-metalworking) | **23** | **2.7 [1.8 – 4.1]** | **1,134** | **4.9 [4.7 – 5.2]** |
| Solvents, fuels | **22** | **2.6 [1.7 – 3.9]** | **339** | **1.5 [1.3 – 1.6]** |
| No information | 13 | 1.5 [0.9 – 2.6] | 203 | 0.9 [0.8 – 1.0] |
| Metals (misc., e.g., coins) | 13 | 1.5 [0.9 – 2.6] | 526 | 2.3 [2.1 – 2.5] |
| Other | 12 | 1.4 [0.8 – 2.5] | 441 | 1.9 [1.8 – 2.1] |
| Tools | 11 | 1.3 [0.7 – 2.3] | 221 | 1.0 [0.8 – 1.1] |
| Plants (non-food) | 9 | 1.1 [0.6 – 2.0] | 419 | 1.8 [1.7 – 2.0] |
| Footwear | 9 | 1.1 [0.6 – 2.0] | 321 | 1.4 [1.3 – 1.6] |
| Perfume, deodorant, aftershave | 8 | 1.0 [0.5 – 1.9] | 368 | 1.6 [1.5 – 1.8] |
| Soap, shampoo, toothpaste | **8** | **1.0 [0.5 – 1.9]** | **917** | **4.0 [3.8 – 4.3]** |
| Dental materials | 8 | 1.0 [0.5 – 1.9] | 259 | 1.1 [1.0 – 1.3] |
| Woods | 8 | 1.0 [0.5 – 1.9] | 286 | 1.2 [1.1 – 1.4] |
| Hairdresser products | **7** | **0.8 [0.4 – 1.7]** | **1,429** | **6.2 [5.9 – 6.6]** |
| Clothing, textiles | **6** | **0.7 [0.3 – 1.5]** | **405** | **1.8 [1.6 – 1.9]** |
| Dental prostheses | 6 | 0.7 [0.3 – 1.5] | 269 | 1.2 [1.0 – 1.3] |
| Leather (other) | **6** | **0.7 [0.3 – 1.5]** | **453** | **2.0 [1.8 – 2.2]** |
| Foods, additives | **5** | **0.6 [0.3 – 1.4]** | **867** | **3.8 [3.5 – 4.0]** |
| Pesticides, herbicides, insecticides | 5 | 0.6 [0.3 – 1.4] | 66 | 0.3 [0.2 – 0.4] |
| Preservatives (biocides) | 4 | 0.5 [0.2 – 1.2] | 94 | 0.4 [0.3 – 0.5] |
| Office supplies | 3 | 0.4 [0.1 – 1.0] | 214 | 0.9 [0.8 – 1.1] |
| Textile furniture | 3 | 0.4 [0.1 – 1.0] | 16 | 0.1 [0.0 – 0.1] |
| Jewelry (non-metal) | 3 | 0.4 [0.1 – 1.0] | 17 | 0.1 [0.0 – 0.1] |
| Animal hair, fur | 2 | 0.2 [0.1 – 0.9] | 107 | 0.5 [0.4 – 0.6] |
| House dust | 2 | 0.2 [0.1 – 0.9] | 124 | 0.5 [0.5 – 0.6] |
| Nail cosmetics | 2 | 0.2 [0.1 – 0.9] | 173 | 0.8 [0.7 – 0.9] |
| Jewelry (metal) | 2 | 0.2 [0.1 – 0.9] | 73 | 0.3 [0.3 – 0.4] |
| Oral medications | 2 | 0.2 [0.1 – 0.9] | 122 | 0.5 [0.4 – 0.6] |
| Medical aids | 2 | 0.2 [0.1 – 0.9] | 125 | 0.5 [0.5 – 0.6] |
| Laboratory chemicals | 2 | 0.2 [0.1 – 0.9] | 85 | 0.4 [0.3 – 0.5] |
| Missing or erroneous entry | 1 | 0.1 [0.0 – 0.7] | 29 | 0.1 [0.1 – 0.2] |
| Implants (metal) | 1 | 0.1 [0.0 – 0.7] | 44 | 0.2 [0.1 – 0.3] |
| Indoor fragrances | 1 | 0.1 [0.0 – 0.7] | 16 | 0.1 [0.0 – 0.1] |
| Furniture | 1 | 0.1 [0.0 – 0.7] | 16 | 0.1 [0.0 – 0.1] |
| Fabric softeners | 1 | 0.1 [0.0 – 0.7] | 28 | 0.1 [0.1 – 0.2] |
| Polishes | 1 | 0.1 [0.0 – 0.7] | 46 | 0.2 [0.2 – 0.3] |

Significant differences are marked in bold. Abbreviations: DGEBA, diglycidyl ether of bisphenol A; OD, occupational dermatitis; 95% CI, 95% confidence interval.

Table S6. Frequencies of patch testing and positive reactions in DGEBA-positive patients with occupational dermatitis (OD), stratified by airborne contact dermatitis and/or face dermatitis.

|  |  | DGEBA-positive OD patients with airborne contact dermatitis and/or face dermatitis (n = 171) | | | |  | DGEBA-positive OD patients without airborne contact dermatitis or face dermatitis (n = 671) | | | |
| --- | --- | --- | --- | --- | --- | --- | --- | --- | --- | --- |
| Substance | Conc. (vehicle) | n tested | n positive | % positive | 95% CI |  | n tested | n positive | % positive | 95% CI |
| Reactive diluents | | | | | | | | | | |
| 1,6-Hexanediol diglycidyl ether (1,6-HDDGE) | 0.25% pet. | 148 | 60 | 40.5 | 32.6 – 48.9 |  | 469 | 209 | 44.6 | 40.0 – 49.2 |
| 1,4-Butanediol diglycidyl ether (1,4-BDDGE) | 0.25% pet. | 148 | 55 | 37.2 | 29.4 – 45.5 |  | 466 | 164 | 35.2 | 30.9 – 39.7 |
| Phenyl glycidyl ether (PGE) | 0.25% pet. | 150 | 53 | 35.3 | 27.7 – 43.5 |  | 487 | 143 | 29.4 | 25.4 – 33.6 |
| p-tert-Butylphenyl glycidyl ether (PTBPGE) | 0.25% pet. | 117 | 39 | 33.3 | 24.9 – 42.6 |  | 379 | 103 | 27.2 | 22.8 – 32.0 |
| Cresyl glycidyl ether (CGE) | 0.25% pet. | 149 | 20 | 13.4 | 8.4 – 20.0 |  | 467 | 74 | 15.8 | 12.7 – 19.5 |
| Butyl glycidyl ether (BGE) | 0.25% pet. | 148 | 15 | 10.1 | 5.8 – 16.2 |  | 470 | 58 | 12.3 | 9.5 – 15.7 |
| Trimethylolpropane triglycidyl ether (TMPTGE) | 0.25% pet. | 137 | 13 | 9.5 | 5.1 – 15.7 |  | 438 | 33 | 7.5 | 5.2 – 10.4 |
|  | | | | | | | | | | |
| Hardeners | | | | | | | | | | |
| m-Xylylene diamine (MXDA) | 0.10% pet. | 138 | 27 | 19.6 | 13.3 – 27.2 |  | 438 | 104 | 23.7 | 19.8 – 28.0 |
| 4,4′-Diaminodiphenylmethane (MDA) | 0.50% pet. | 149 | 20 | 13.4 | 8.4 – 20.0 |  | 489 | 70 | 14.3 | 11.3 – 17.7 |
| Isophorone diamine (IPDA) | 0.50% pet. | 150 | 14 | 9.3 | 5.2 – 15.2 |  | 473 | 42 | 8.9 | 6.5 – 11.8 |
| 2,4,6-Tris(dimethylaminomethyl)phenol (tris-DMP) | 0.50% pet. | 39 | 3 | 7.7 | 1.6 – 20.9 |  | 139 | 11 | 7.9 | 4.0 – 13.7 |
| Trimethylhexane-1,6-diamine (TMHDA) | 0.50% pet. | 139 | 9 | 6.5 | 3.0 – 11.9 |  | 438 | 28 | 6.4 | 4.3 – 9.1 |
| Diethylenetriamine (DETA) | 1% pet. | 25 | 1 | 4.0 | 0.1 – 20.4 |  | 109 | 3 | 2.8 | 0.6 – 7.8 |
| Triethylenetetramine (TETA) | 0.50% pet. | 149 | 3 | 2.0 | 0.4 – 5.8 |  | 462 | 23 | 5.0 | 3.2 – 7.4 |
| Ethylenediamine dihydrochloride (EDHCl) | 1% pet. | 86 | 0 | 0.0 | 0.0 – 4.2 |  | 439 | 4 | 0.9 | 0.2 – 2.3 |
|  | | | | | | | | | | |
| Other epoxy-related substances | | | | | | | | | | |
| Bisphenol F epoxy resin (DGEBF) | 0.25% pet. | 5 | 4 | 80.0 | 28.4 – 99.5 |  | 29 | 17 | 58.6 | 38.9 – 76.5 |
| Bisphenol A-glycidyl methacrylate (BIS-GMA) | 2% pet. | 141 | 36 | 25.5 | 18.6 – 33.6 |  | 427 | 74 | 17.3 | 13.9 – 21.3 |

For CAS numbers, see Table S1. Significant differences are marked in bold; none were observed in this table. Abbreviations: pet., petrolatum; OD, occupational dermatitis; 95% CI, 95% confidence interval.
